# Serum levels of interleukin-17, -18, -22, and -25 in patients with bullous pemphigoid before and after treatment

**DOI:** 10.3389/fmed.2025.1559372

**Published:** 2025-07-29

**Authors:** Xinyi Hong, Xiuqin Wang, Yin Cheng, Hanqing Song, Congcong Xu, Peiguang Wang

**Affiliations:** ^1^Department of Dermatology, First Affiliated Hospital of Anhui Medical University, Hefei, China; ^2^Institute of Dermatology, Anhui Medical University, Hefei, China; ^3^Key Laboratory of Dermatology (Anhui Medical University), Ministry of Education, Hefei, China; ^4^Collaborative Innovation Center of Complex and Severe Skin Disease, Anhui Medical University, Hefei, China; ^5^Wuxi Ninth People's Hospital, Soochow University, Wuxi, China

**Keywords:** IL-17, IL-18, IL-22, IL-25, EOS, BP180 antibodies, BP230 antibodies, bullous pemphigoid

## Abstract

**Objective:**

This study aimed to explore the relationship between serum levels of interleukin-17 (IL-17), interleukin-18 (IL-18), interleukin-22 (IL-22), interleukin-25 (IL-25), anti-BP180 antibodies, anti-BP230 antibodies, and immunoglobulin E (IgE) and the percentage of eosinophils (EOS) in peripheral blood and disease activity and severity in bullous pemphigoid (BP) patients.

**Methods:**

Blood samples from 61 BP inpatients were collected on the first day of admission and again 1 week after systemic corticosteroid treatment. Additionally, blood specimens were collected from 61 healthy controls. The concentrations of IL-17, IL-18, IL-22, IL-25, IgE, and anti-BP180 or anti-BP230 antibodies were measured using ELISA kits. Various statistical methods were used, including the Wilcoxon test, multifactorial logistic regression, ROC survival curve, and Spearman’s correlation analysis.

**Results:**

The mean serum levels of IL-17, IL-18, IL-22, IL-25, and EOS percentage in BP patients were higher than those in healthy controls (all *p* < 0.001), and all of these levels decreased after treatment (all *p* < 0.001). There was no statistically significant difference in the titer of anti-BP180 or anti-BP230 antibodies before and after treatment. A binary multivariate logistic regression analysis indicated the statistically significant effect of IL-18, IL-25, or EOS on BP (*p* < 0.001). Spearman’s correlation analysis revealed that the serum levels of IL-17, IL-22, or IL-25 were all strongly correlated with the involved surface area (*p* < 0.001). In addition, the serum level of IL-17 and the percentage of EOS were associated with the titer of anti-BP180 antibodies (*p* < 0.05).

**Conclusion:**

IL-17, IL-18, IL-22, IL-25, or EOS may be involved in the development of BP, and can be used as biological indicators for monitoring disease severity.

## 1 Introduction

Bullous pemphigoid (BP) is the most common autoimmune subepidermal bullous disorder, mainly affecting the elderly (1, 2). Some studies have shown that Th17 cells are involved in the pathogenesis of this disease (3–5). Th17 cells secrete various inflammatory mediators, including IL-17, IL-22, IL-25, TNF-α, granulocyte-macrophage colony-stimulating factor (GM-CSF), neutrophil-recruiting chemokines, and antimicrobial peptides, all of which lead to tissue damage (6). Arakawa et al. (7) found that the serum level of IL-17 increased in the lesional skin and blister fluid. Interestingly, Plée et al. (8) discovered that relapsing patients with BP showed a continuously increasing serum IL-17 concentration during the first month of treatment. Therefore, they proposed that the longitudinal measurement of IL-17 in serum can predict relapse in BP patients. Moreover, circulating IgE is elevated in up to 85% of BP patients, and increased levels of both EOS and IgE have been associated with poor prognosis (9, 10). In this study, we aim to explore the relationship between serum levels of IL-17, IL-22, IL-25, EOS in peripheral blood, anti-BP180 antibodies, anti-BP230 antibodies, and IgE and disease activity and severity in patients with bullous pemphigoid.

## 2 Materials and methods

### 2.1 Patients and data collection

Clinical data and blood samples were randomly collected from 61 BP inpatients and 61 healthy controls. Among the BP patients, the mean age was 67.84 ± 13.71 years, and the BP group included 27 men and 34 women. The course of the disease ranged from 4 days to 10 years. The mean interval between two blood draws before and after treatment with systemic corticosteroids was 7.50 ± 2.20 days.

### 2.2 Laboratory evaluation

All serum indicators were measured using the enzyme-linked immunosorbent assay (ELISA) sandwich technique. Kits for IL-17, IL-18, IL-22, and IL-25 were purchased from Shanghai Hengyuan Biotechnology Co., while IgE kits were obtained from the German EU Medical Laboratory Diagnosis Corporation. Anti-BP180 and anti-BP230 antibody kits were purchased from Medical Biology Laboratory, Japan Co. All assays were performed in strict accordance with the manufacturers’ instructions.

### 2.3 Statistical analysis

Statistical analyses were performed using SPSS version 27.0. Quantitative data were first analyzed to evaluate whether these data conform to a normal distribution. We performed a t-test and a one-way ANOVA to ensure the data met a normal distribution. On the contrary, if data did not meet the assumptions of normality, then non-parametric tests were used. Patients and controls were treated as two independent samples, and the Wilcoxon test was performed. Values from the BP patients before and after treatment were treated as paired samples and analyzed using the rank-sum test. The chi-squared test was used to analyze qualitative data. The presence or absence of BP was considered a binary outcome. All collected confounding factors, including age and gender, were included in a multivariate logistic regression analysis to calculate adjusted odds ratios (ORs) or coefficients. This allowed us to identify indicators significantly correlated with the increase in BP serum levels. ROC survival analysis was then used to evaluate the respective diagnostic efficiency and to obtain the truncation value. A combined diagnostic indicator was constructed by creating a comprehensive predictive variable through logistic regression, followed by an ROC analysis. Spearman’s correlation analysis was used to compare the correlation of these indicators with the area of lesions involved.

## 3 Results

### 3.1 Demographics and clinical characteristics of patients

The demographic characteristics of the 61 patients and 61 healthy controls are shown in Table 1. We compared the age and gender of the two groups. There were no significant differences between the two groups (*p* > 0.05, Table 1). Among the 61 patients with BP, several had coexisting chronic conditions, including 3 with psoriasis, 15 with hypertension, 8 with diabetes, 8 with cerebral infarctions, 1 with hypothyroidism, 1 with rheumatoid arthritis, and 1 with vitiligo.

**Table 1:** Demographics characteristics of patients.

	BP group (*n* = 61)	Healthy control group (*n* = 61)	*p*-value
Age (years)	67.84 ± 13.71	66.21 ± 11.482	0.445
Sex			
Male	27	25	0.134
Female	34	36	

Data are mean ± standard, *n* (%). *p*-values were calculated using an independent samples *t*-test, *χ*^2^ test.*p-value < 0.05.

### 3.2 Cytokine levels and eosinophil numbers

The mean serum level of cytokines or the EOS percentage of BP patients was all higher than that of healthy controls, and the differences were statistically significant (all *p* < 0.001). The serum levels of cytokines, EOS, and IgE after treatment were strikingly lower than those before treatment in BP patients, and the difference was statistically significant (all *p* < 0.001). However, there were no statistically significant differences in the titer of anti-BP180 or anti-BP230 antibodies before and after treatment (Table 2).

**Table 2:** Cytokine levels and eosinophil numbers.

Indicators	BP group/before treatment (*n* = 61)	Healthy control group (*n* = 61)	*p*-value	After treatment	*p*-value
IL-17 (ng/L)	46.16 (40.51, 56.83)	15.32 (12.99, 17.40)	<0.001	38.26 (32.53, 50.53)	<0.001
IL-18 (ng/L)	111.66 (95.31, 134.80)	48.70 (41.24, 69.19)	<0.001	100.89 (81.64, 115.12)	<0.001
IL-22 (ng/L)	34.71 (29.83, 49.18)	16.71 (13.65, 18.84)	<0.001	27.90 (25.06, 42.81)	<0.001
IL-25 (ng/L)	51.14 (40.42, 70.46)	24.85 (21.55, 27.61)	<0.001	42.21 (27.10, 64.05)	<0.001
EOS (%)	7.81 (3.85, 16.71)	1.00 (0.26, 1.87)	<0.001	0.22 (0.04, 1.04)	<0.001
BP180 (u/mL)	285.53 (104.36, 428.52)			318.08 (115.20, 422.31)	0.391
BP230 (u/mL)	6.22 (1.73, 18.69)			4.06 (1.64, 38.61)	0.814
IgE (mg/mL)	1141.08 (954.62, 1401.62)			964.19 (815.43, 1283.06)	0.024

Data are median (IQR). *p*-values are calculated using the Wilcoxon test. *p-value < 0.05.

### 3.3 Logistic regression

We included 61 BP patients with complete data for all variables in the binary logistic regression model via stepwise regression. We added the collected confounding factors in the multivariate logistic regression analysis, which included age and gender. We found that the serum levels of IL-18 (OR = 1.153, 95%CI:1.055–1.259, *p* = 0.002), IL-25 (OR = 1.880, 95%CI:1.337–2.644, *p* < 0.001), and EOS (OR = 3.153, 95%CI:1.421–6.995, *p* = 0.005) were significantly associated with the presence of BP serum (Table 3).

**Table 3:** Logistic regression analysis for the related factors predicting BP.

Indicators	B	SR	Wald	*p-*value	OR	OR 95%CI	
						Lower limit	Upper limit
IL-17 (ng/L)	2.928	227.728	0.000	0.990	18.689	0	1.299E+195
IL-18 (ng/L)	0.142	0.045	9.939	0.002	1.153	1.055	1.259
IL-22 (ng/L)	6.858	456.596	0.001	0.988	951.157	0	–
IL-25 (ng/L)	0.631	0.174	13.160	<0.001	1.880	1.337	2.644
EOS (%)	0.583	0.407	7.980	0.005	3.153	1.421	6.995

B, partial regression coefficients; S. E., standard error; ORs, odds ratios; CI, confidence interval.

### 3.4 ROC curves

**Figure 1:**
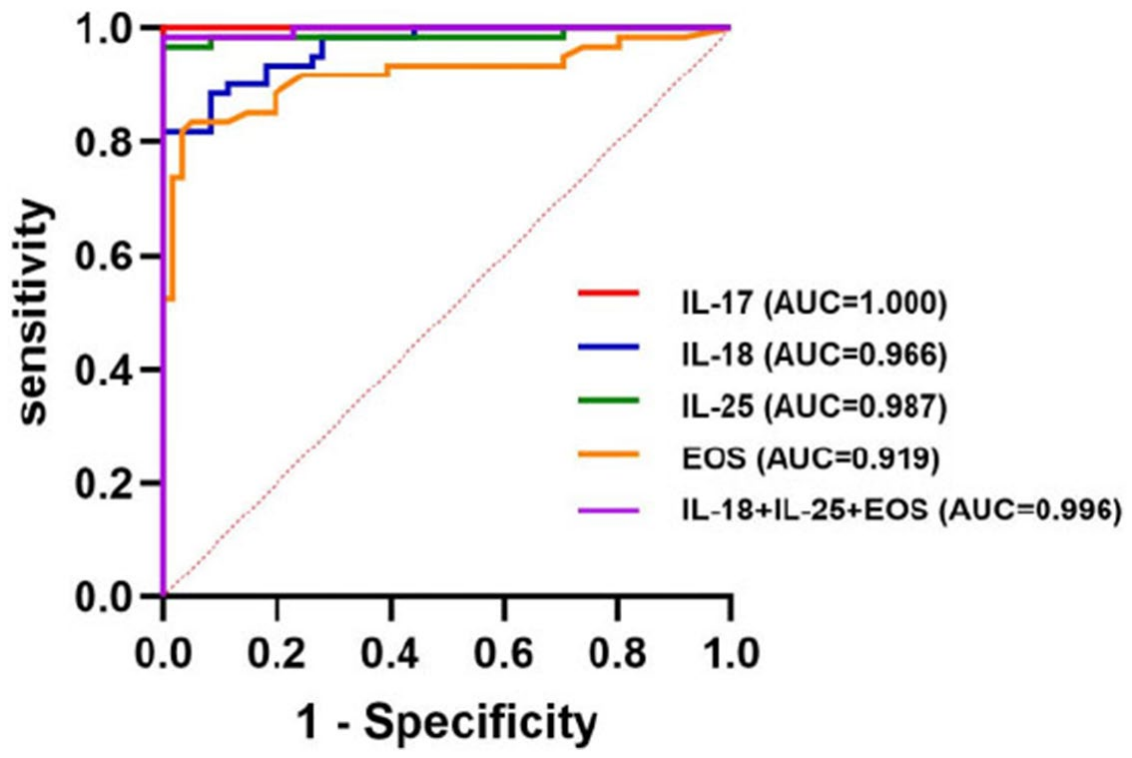
ROC curves of logistic model.

ROC curves were used to evaluate the predictive value of the binary logistic model (Figure 1). The area under the ROC curve (AUC) for the serum levels of IL-18, IL-25, and EOS were 0.966 (95% CI: 0.940–0.992, *p* < 0.001), 0.987 (95% CI: 0.964–1.000, *p* < 0.001), and 0.919 (95% CI: 0.864–0.973, *p* < 0.001), respectively. The corresponding sensitivities were 82, 96.7, and 82.8%, and the Youden indices were 0.820, 0.967, and 0.787, respectively. Their associated threshold values were 89.485 ng/L, 32.625 ng/L, and 3.135%, respectively. The combined diagnostic efficiency of these three indicators yielded an AUC of 0.996. Additionally, we included the ROC curve for the serum level of IL-17, which showed an AUC value of 1, with 100% sensitivity, a cutoff value of 25.540 ng/L, and a *p*-value of < 0.0001 (Table 4; Figure 1).

**Table 4:** AUC, sensitivity, Youden index_max_, and threshold of ROC curve analysis.

Indicators	AUC	Sensitivity	Youden’s Index_max_	Threshold	*p-*value
IL-17 (ng/L)	1.000	100%	1.000	25.540	<0.0001
IL-18 (ng/L)	0.966(0.940–0.992)	82.00%	0.820	89.485	<0.001
IL-25 (ng/L)	0.987(0.964–1.000)	96.70%	0.967	32.625	<0.001
EOS (%)	0.919(0.864–0.973)	82.80%	0.787	3.135	<0.001
IL-18 + IL-25 + EOS	0.996(0.989–1.000)	98.40%	0.984	113.395	<0.001

AUC, the area under the ROC curve; Youden’s index, sensitivity+specificity −1; threshold, The corresponding indicator value of Youden index_max._

### 3.5 BP-related indicators with the involved area of lesions

Spearman’s correlation analysis showed that the serum levels of IL-17 (*r* = 0.787), IL-22 (*r* = 0.749), and IL-25 (*r* = 0.743) were strongly correlated with disease severity, but the levels of IL-18 (*r* = 0.287) and the titer of anti-BP180 antibody (*r* = 0.258) were weakly correlated with disease severity (Table 5).

**Table 5:** BP-related indicators with the involved area of lesions.

Indicators	*R*	*p-*value
IL-17 (ng/L)	0.787	<0.001
IL-18 (ng/L)	0.287	0.025
IL-22 (ng/L)	0.749	<0.001
IL-25 (ng/L)	0.743	<0.001
EOS (%)	0.198	0.127
BP180 (u/mL)	0.258	0.046
BP230 (u/mL)	0.131	0.320
IgE (mg/mL)	0.175	0.280

### 3.6 Correlation analysis of BP-related factors

The serum level of IL-17 was correlated with the levels of IL-22 (*r* = 0.661), IL-25 (*r* = 0.758), and anti-BP180 antibody (*r* = 0.272). The serum level of IL-18 was correlated with that of IL-22 (*r* = 0.329). In addition to the serum levels of IL-17 and IL-18, the serum level of IL-22 was strongly correlated with that of IL-25 (*r* = 0.684). Meanwhile, the serum level of EOS (*r* = 0.311) was correlated with the titer of anti-BP180 antibody (*p* < 0.05) (Table 6).

**Table 6:** Correlation analysis of BP-related indicators (*r*/*p*).

	IL-17	IL-18	IL-22	IL-25	EOS	BP180	BP230	IgE
IL-17	–	0.238 (*p* = 0.064)	0.661 (*p* < 0.001)	0.758 (*p* < 0.001)	0.070 (*p* = 0.592)	0.272 (*p* = 0.035)	0.059 (*p* = 0.653)	0.026 (*p* = 0.876)
IL-18	0.238 (*p* = 0.064)	–	0.329 (*p* = 0.010)	0.182 (*p* = 0.160)	−0.227 (*p* = 0.078)	0.167 (*p* = 0.203)	0.001 (*p* = 0.996)	0.249 (*p* = 0.122)
IL-22	0.661 (*p* < 0.001)	0.329 (*p* = 0.010)	–	0.684 (*p* < 0.001)	0.191 (*p* = 0.141)	0.176 (*p* = 0.179)	0.107 (*p* = 0.414)	0.110 (*p* = 0.498)
IL-25	0.758 (*p* < 0.001)	0.182 (*p* = 0.160)	0.684 (*p* < 0.001)	–	0.131 (*p* = 0.316)	0.076 (*p* = 0.564)	0.102 (*p* = 0.439)	−0.093 (*p* = 0.567)
EOS	0.070 (*p* = 0.592)	−0.227 (*p* = 0.078)	0.191 (*p* = 0.141)	0.131 (*p* = 0.316)	–	0.311 (*p* = 0.016)	0.105 (*p* = 0.423)	0.193 (*p* = 0.233)
BP180	0.272 (*p* = 0.035)	0.167 (*p* = 0.203)	0.176 (*p* = 0.179)	0.076 (*p* = 0.564)	0.311 (*p* = 0.016)	–	0.099 (*p* = 0.451)	0.136 (*p* = 0.408)
BP230	0.059 (*p* = 0.653)	0.001 (*p* = 0.996)	0.107 (*p* = 0.414)	0.102 (*p* = 0.439)	0.105 (*p* = 0.423)	0.099 (*p* = 0.451)	–	0.293 (*p* = 0.070)
IgE	0.026 (*p* = 0.876)	0.249 (*p* = 0.122)	0.110 (*p* = 0.498)	−0.093 (*p* = 0.567)	0.193 (*p* = 0.233)	0.136 (*p* = 0.408)	0.293 (*p* = 0.070)	–

### 3.7 BP-related indicators: serum levels for different durations

**Figure 2:**
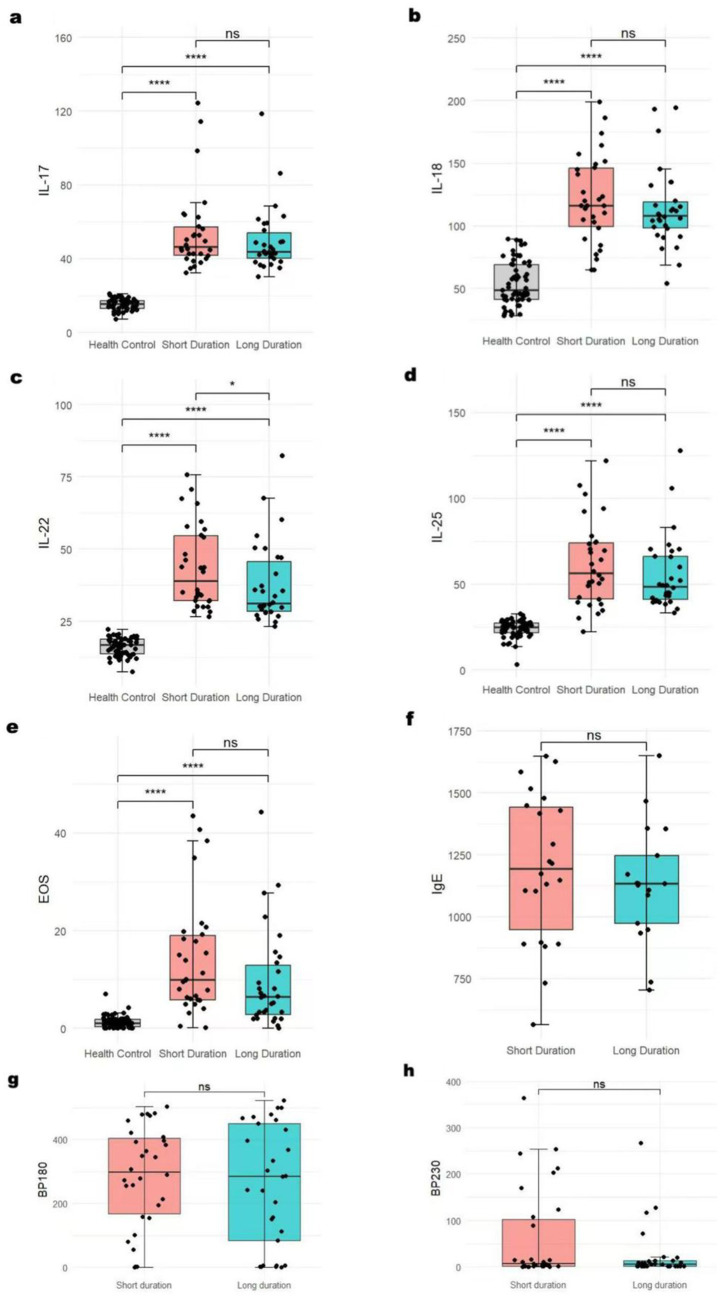
BP-related indicators’ serum levels in different durations *p < 0.05; **p < 0.01; ***p < 0.001; ****p < 0.0001; ns: no significance. The serum IL-22 **(c)** level showed statistically significant differences among different durations. The levels of IL-17 **(a)**, IL-18 **(b)**, IL-25 **(d)**, EOS **(e)**, IgE **(f)**, and BP180/230 **(g,h)** did not show any differences.

Taking the median duration of the disease (93 days) as the dividing line, patients with BP were divided into two groups: the long-duration group and the short-duration group. We found that, except for the serum IL-22 level, which showed statistically significant differences among different durations, the levels of other indicators did not show any differences (Figure 2).

## 4 Discussion

IL-17 originates from several inflammatory cells, including neutrophils, dendritic cells, group 3 innate lymphoid cells (ILC3s), NK cells, γδ T cells, and CD4^+^T cells (11). Chakiebska et al. (12) found high numbers of IL-17a + CD4 + lymphocytes in the peripheral blood of BP patients. IL-17 is pathogenically relevant in BP, and its serum level was positively associated with disease severity (11, 12). Interestingly, in another part of the study on patients with BP, only the serum level of IL-17 was found to be elevated, but it was not related to the severity of the disease (13, 14). Furthermore, some studies suggest that IL-17 can cause eosinophilia, thereby leading to skin inflammation (15, 16). Our study showed that the serum level of IL-17 was markedly increased, and its expression was strikingly decreased after treatment in BP patients. Moreover, a strong positive correlation was observed between the serum level of IL-17 and the involved area of BP lesions. The serum level of IL-17 is correlated with the levels of IL-22, IL-25, and the autoantibodies of BP180. This finding suggests that these biological indicators may interact with each other in the occurrence of BP. Our results support the fact that IL-17 is involved in the complex pathogenesis of BP, and it is very likely to be related to the severity of the disease. It is worth noting that an increasing number of experts have reported that IL-17 inhibitors can both treat and induce BP when used in the treatment of immune-related diseases (17). This finding suggests that IL-17 plays a significant and complex role in the occurrence and resolution of BP. Our research findings can further clarify the potential role of IL-17 in the pathogenesis of BP and support the use of proper IL-17 inhibition for the treatment of BP.

IL-18 belongs to the IL-1 family and can promote the production of Th2/Th17 cytokines (18). Its level was remarkably elevated in BP patients (19–21). Similarly, we also found a significant increase of IL-18 in the sera of BP patients. Although one study indicated a significant correlation between serum IL-18 levels and the titer of anti-BP180 antibodies, our study found no significant correlation between the two indicators (20).

IL-22 is derived from Th17 cells, group 3 innate lymphoid cells (ILC3s), and other immune cells, and it is involved in diverse inflammatory and autoimmune conditions (22). A synergistic effect exists between IL-22 and IL-25 (23). Our investigation revealed that the serum level of IL-22 was significantly increased but markedly reduced after treatment in BP patients. In addition, its serum level might be strongly correlated with the severity of BP.

IL-25 is secreted from T cells, dendritic cells, group 2 innate lymphoid cells, or epithelial cells (24). IL-25 promotes the production of cytokines, IgE, and eosinophilia. It can also activate nuclear factor kappa B (NF-kB), mitogen-activated protein kinases (MAPKs), and Janus kinase/signal transducer and activator of transcription (JAK/STAT) (25, 26). In this study, the serum level of IL-25 was significantly increased, and its level decreased after treatment. Furthermore, the level of IL-25 was strongly associated with the severity of the disease, similar to the levels of IL-17 and IL-22.

Spearman’s correlation analysis revealed that the serum level of IL-17 was correlated with the levels of IL-22, IL-25, and anti-BP180 antibodies. The serum level of IL-18 was correlated with that of IL-22. In addition to IL-17 and IL-18, the serum level of IL-22 was strongly correlated with IL-25. Moreover, the serum level of EOS was correlated with the titer of anti-BP180 antibodies. These findings may provide clues about how these biological indicators function within the development of BP.

The level of anti-BP180 autoantibodies reflects the severity of the disease in BP. Some studies found that the level of anti-BP180 autoantibodies significantly decreased after the lesion completely recovered following treatment (27–29). In our study, the values of indicators such as IL-17 significantly decreased after treatment. The level of anti-BP180/230 antibodies showed no significant difference before and after treatment. We consider this to be related to the short monitoring time of the treatment, and the change in antibody levels lagged behind the changes in cytokines and inflammatory cells. During the onset of BP, the target antigen BP180/230 activates auto-reactive CD4 + T cells (especially the Th2/Th17 subgroups), and T cells provide co-stimulatory signals and cytokines such as IL-17 to assist in the activation and differentiation of B cells, thereby generating anti-BP180/230 autoantibodies. The production of anti-BP180/230 autoantibodies is related to IL-17. The level of IL-17 may be more helpful for the early diagnosis and disease monitoring of BP than the level of anti-BP180 antibodies.

In summary, these findings suggest that IL-17, IL-18, IL-22, IL-25, and EOS are closely related to the occurrence of BP. Therefore, it is beneficial to evaluate the severity and progression of this disease by monitoring these cytokines and the EOS count. Selective inhibition of these cytokines may effectively control the progression of BP. Galluzzo et al. (30) reported that brodalumab for psoriasis antagonizes signaling from IL-25, IL-17A, IL-17F, and IL-17A/F by blocking the IL-17 receptor A, which provides a new idea for the treatment of BP.

## 5 Limitations

Our study has several limitations. First, it was a single-center study. Our sample size is not large enough. Second, we only collected blood samples from BP inpatients on the first day of admission and approximately 1 week after the treatment of systemic corticosteroids. It was challenging to obtain the levels of serum biological indicators before the onset of the disease in these hospitalized patients. Therefore, we were unable to determine the causal relationship between these factors and the occurrence of BP. We could only state that there is a correlation between the two. In addition, the relatively small sample size of patients used to develop a prediction model might be associated with selection bias, reduce statistical power, and affect the reliability of the conclusions drawn. When interpreting and applying the results of our research, caution is necessary. Carrying out a larger prospective study that includes more covariates and reduces collinearity among study factors would be of greater significance. Finally, we only measured the lesion area of BP but did not calculate the BPDAI score. The lesion area alone cannot accurately represent the severity of the disease. BPDAI is a standardized tool used to assess the activity and severity of bullous pemphigoid. It consists of two parts: skin lesions and mucosal lesions (31).

## 6 Conclusion

In conclusion, these four cytokines and EOS are closely linked to both the progression and severity of BP and may serve as suitable candidates for potential prognostic indicators of BP. Further investigation with a larger cohort is essential to verify the correlation between these markers and BP.

## Data Availability

The original contributions presented in the study are included in the article/Supplementary material, further inquiries can be directed to the corresponding author.
